# Cancers in the TREAT Asia HIV Observational Database (TAHOD): a retrospective analysis of risk factors

**DOI:** 10.1186/1758-2652-13-51

**Published:** 2010-12-10

**Authors:** Kathy Petoumenos, Eugenie Hui, Nagalingeswaran Kumarasamy, Stephen J Kerr, Jun Yong Choi, Yi-Ming A Chen, Tuti Merati, Fujie Zhang, Poh-Lian Lim, Somnuek Sungkanuparph, Sanjay Pujari, Sasheela Ponnampalavanar, Rosanna Ditangco, Christopher KC Lee, Andrew Grulich, Matthew G Law

**Affiliations:** 1 National Centre in HIV Epidemiology and Clinical Research, University of New South Wales, Sydney, NSW, Australia; 2 Queen Elizabeth Hospital, Hong Kong, China; 3 YRG Centre for AIDS Research and Education, Chennai, India; 4 HIV-Netherlands Australia Thailand Research Collaboration, 104 Ratchadamri Road, Bangkok 10330. Thailand; 5 Department of Internal Medicine and AIDS Research Institute, Yonsei University College of Medicine, Seoul, South Korea; 6 Taipei Veterans General Hospital and AIDS Prevention and Research Centre, National Yang-Ming University, Taipei, Taiwan; 7 Faculty of Medicine Udayana University & Sanglah Hospital, Bali, Indonesia; 8 Beijing Ditan Hospital, Beijing, China; 9 Tan Tock Seng Hospital, Singapore; 10 Faculty of Medicine Ramathibodi Hospital, Mahidol University, Bangkok, Thailand; 11 Institute of Infectious Diseases, Pune, India; 12 University Malaya Medical Centre, Kuala Lumpur, Malaysia; 13 Research Institute for Tropical Medicine, Manila, Philippines; 14 Hospital Sungai Buloh, Kuala Lumpur, Malaysia

## Abstract

**Background:**

This retrospective survey describes types of cancers diagnosed in HIV-infected subjects in Asia, and assesses risk factors for cancer in HIV-infected subjects using contemporaneous HIV-infected controls without cancer.

**Methods:**

TREAT Asia HIV Observational Database (TAHOD) sites retrospectively reviewed clinic medical records to determine cancer diagnoses since 2000. For each diagnosis, the following data were recorded: date, type, stage, method of diagnosis, demographic data, medical history, and HIV-related information. For risk factor analyses, two HIV-infected control subjects without cancer diagnoses were also selected. Cancers were grouped as AIDS-defining cancers (ADCs), and non-ADCs. Non-ADCs were further categorized as being infection related (NADC-IR) and unrelated (NADC-IUR).

**Results:**

A total of 617 patients were included in this study: 215 cancer cases and 402 controls from 13 sites. The majority of cancer cases were male (71%). The mean age (SD) for cases was 39 (10.6), 46 (11.5) and 44 (13.7) for ADCs, NADC-IURs and NADCs-IR, respectively. The majority (66%) of cancers were ADCs (16% Kaposi sarcoma, 40% non-Hodgkin's lymphoma, and 9% cervical cancer). The most common NADCs were lung (6%), breast (5%) and hepatocellular carcinoma and Hodgkin's lymphoma (2% each). There were also three (1.4%) cases of leiomyosarcoma reported in this study. In multivariate analyses, individuals with CD4 counts above 200 cells/mm^3 ^were approximately 80% less likely to be diagnosed with an ADC (p < 0.001). Older age (OR: 1.39, p = 0.001) and currently not receiving antiretroviral treatment (OR: 0.29, p = 0.006) were independent predictors of NADCs overall, and similarly for NADCs-IUR. Lower CD4 cell count and higher CDC stage (p = 0.041) were the only independent predictors of NADCs-IR.

**Conclusions:**

The spectrum of cancer diagnoses in the Asia region currently does not appear dissimilar to that observed in non-Asian HIV populations. One interesting finding was the cases of leiomyosarcoma, a smooth-muscle tumour, usually seen in children and young adults with AIDS, yet overall quite rare. Further detailed studies are required to better describe the range of cancers in this region, and to help guide the development of screening programmes.

## Background

HIV infection is associated with an increased risk of a range of cancers, including Kaposi sarcoma (KS), non-Hodgkin's lymphoma (NHL), and cervical cancer [1-3], which are designated as AIDS-defining cancers (ADCs) [4]. Cohort studies of people with HIV have consistently reported an increased risk for non-AIDS-defining cancers (NADCs), such as Hodgkin's disease, and anogenital cancers [1,3,5-10]. However, the epidemiology of cancer in HIV-infected people continues to evolve [11,12], particularly since the introduction of highly active antiretroviral therapy (HAART), which has led to significantly improved survival after HIV diagnosis [13-20].

The widespread use of HAART has resulted in decreases in the incidence of KS and NHL [11,21], although a decline in incidence for other cancers is less evident [11]. Additionally, as patients with HIV are living longer, malignancy is becoming an increasingly prominent cause of death [12,22-25]. Increasingly reported NADCs include lung cancer, liver cancer, anal cancer and leukaemia.

There are limited data on cancer occurrence in HIV-infected patients in Asia. Investigators at the Ramathibodi Hospital at Mahidol University, Bangkok, Thailand, a collaborating site of the Therapeutics Research, Education and AIDS Training in Asia (TREAT Asia) HIV Observational Database (TAHOD), have retrospectively reviewed pathological reports and medical records on malignancies and treatment outcome in Thai HIV-infected patients. Between 1999 and 2003, 3% of more than 1100 HIV-patients were diagnosed with malignancies. More than half (62%) were ADCs, NHL being the most common. NADCs included breast, colorectal and lung cancer.

In this study, treatment of the malignancy was the only significant factor associated with survival, while age, prior AIDS diagnosis and antiretroviral treatment history were not [26]. In India, among all cancers reported at the Tata Memorial Hospital in Mumbai from 2001 to 2005, 251 cases were identified to be in HIV-positive people, and more than half (56%) were NADCs. Among the ADCs, NHL was the most common, and there were no cases of KS. Among the NADCs, head and neck cancers were the most common [27].

Insight into the patterns of cancer occurrence in HIV/AIDS can be inferred from studies of cancer-identifying risk factors in other immune-deficient populations. Such populations include organ transplant recipients who undergo iatrogenic immune suppression post-transplantation. A recent large study of cancer occurrence in Australian kidney transplant recipients found a marked increase in cancer risk at a wide variety of sites. After transplantation, 25 cancer sites occurred at significantly increased incidence, and risk increased three-fold at 18 of these sites. Most of these cancers were of known or suspected viral aetiology. These data suggest a broader than previously appreciated role of the interaction between the immune system and common viral infections in the aetiology of cancer [28,29].

Our objective was to undertake a retrospective survey of cancer diagnoses in HIV-infected subjects at the clinical sites in Asia that currently participate in the TREAT Asia HIV Observational Database (TAHOD). The specific aims of this study were to describe the range of cancers diagnosed in HIV-infected subjects in Asia, and to determine risk factors for cancer in HIV-infected subjects in Asia compared with contemporaneous HIV-infected subjects without cancer.

## Methods

TAHOD commenced in 2003 and is a collaborative observational cohort study including 17 participating clinical sites in the Asia and Pacific region. A detailed description of this collaboration has been published previously [30]. TAHOD sites that maintained patient visit records from 2000 onwards were invited to participate in this retrospective case-control study. Individual TAHOD sites determined their capacity to review their entire clinic records for cases, or whether they restrict the review to TAHOD only patients.

In total, 13 of the 17 TAHOD sites were able to participate. Eight of the sites reviewed all clinic patient records regardless of whether patients were enrolled in the TAHOD study (n = 7 records from 2000; n = 1 record from 2004), totalling an estimated 12,000 patients. Four sites limited record reviews to patients within the TAHOD study (n = 3) or those participating in clinical trials (n = 1), approaching more than 800 patient records. In total, an estimated 13,000 patient records were reviewed to ascertain cancer cases. One site was not able to recruit controls.

Ethical approval for TAHOD was obtained from the University of New South Wales, Sydney, Australia, and for individual clinical sites from their local institutional review boards, as required. Unless required by a site's local ethics, written consent was not a requirement of sites in TAHOD because data are collected in an anonymous form. All TAHOD study procedures were developed in accordance with the revised 1975 Helsinki Declaration

### Selection of cases

Contributing sites were required to review all medical records from 1 January 2000 (or later, if relevant) to 1 January 2008 to ascertain cases of cancer diagnosed. Only the first cancer diagnosed was considered for each case.

To standardize ascertainment and reporting across the sites, a half-day, face-to-face investigator training session was conducted. The facilitators of this training session were cancer epidemiologists from the National Centre in HIV Epidemiology and Clinical Research (NCHECR), and the Lowy Cancer Research Centre, University of New South Wales. The training included issues of determining morphology (histologocial classification of the cancer tissue, site and staging of the cancer, as well as establishing a date of diagnosis). The training also included describing the sources and hierarchy of information for a cancer diagnosis (e.g., pathology, biopsy, or cytology report, laboratory data, imaging, treatment details, autopsy report).

Participating sites were advised to report all pathological diagnoses of invasive/malignant, *in situ *or unknown/uncertain neoplasms. In the absence of histologic or cytologic confirmation, sites were advised to report a case based on a clinical diagnosis made by a recognized medical practitioner.

### Selection of controls

For each case, two contemporaneous cancer-free, HIV-positive controls were selected from a complete list of patients attending the respective clinic on the day or corresponding week that the cancer case was first diagnosed. If both daily and weekly patient lists were available, then sites randomly selected two controls from the daily patient list. Instructions were provided to the sites to ensure a standardized approach for the selection of cases and control. The selection of controls was determined using the RANDBETWEEN function in Excel.

### Data collection

Data were entered into an Access database developed at the NCHECR. The data were then forwarded to the NCHECR for case review and confirmation. The following data were obtained from patient medical records and reported for both cases and controls: date of birth (or age); sex; mode of HIV exposure (patient self-report); date of first positive HIV test; ethnicity; hepatitis B (HBV) and hepatitis C (HCV) status (defined as HBV surface antigen positive and HCV antibody positive, respectively); AIDS-defining illness diagnosed prior to case diagnosis; CDC stage; CD4 cell count at diagnosis; smoking and alcohol use (patient self-report); antiretroviral treatment history; and date of death (if known).

Case data included: date of diagnosis; site; morphology; method of diagnosis (e.g., pathology, cytology, radiology, laboratory data, clinical diagnosis, death certificate); stage; node; and class scheme. All measures (excluding death, patient demographics and cancer treatment) were recorded at the time of the cancer diagnosis for the case, or at the time of the corresponding clinic visit for the control.

### Case validation

Cancer cases were reviewed by a medical cancer epidemiologist at NCHECR, and clarification was sought from the sites as needed.

### Data analysis

All cancers (excluding *in situ *neoplasms) were categorized into the following groups based on published reports (29-31): ADCs (KS, NHL and cervical cancer); NADCs infection-unrelated (NADCs-IUR); and NADCs infection-related (possible/probable) (NADCs-IR). NADCs-IR included: hepatocellular carcinoma, Hodgkin's lymphoma, leiomyosarcoma, and cancers of the anus, bladder, larynx, nasopharynx, oral cavity, penis, stomach, tongue and tonsils [29,31,32]. Key baseline demographic, HIV disease stage and health status were also summarized. Baseline was defined as the date on which the cases were first diagnosed with cancer, and for the controls, the date on which the control attended the clinic (on the day of, or within one week of the matched cases diagnosis date).

### Statistical analysis

Conditional logistic regression methods were used to determine factors associated with ADCs and NADCs. The following baseline demographic and clinical factors were assessed as covariates: age; mode of HIV exposure; ethnicity; prior AIDS; CDC stage; CD4 cell count (within one year prior to case diagnosis); HBV and HCV status; antiretroviral treatment history; and smoking and alcohol use.

A sensitivity analysis for the NADC-IR endpoint was also conducted, excluding bladder, larynx and oral cavity cancers, less than 20% of which had been attributed to infections. All covariates with p < 0.100 in univariate analyses were assessed in the multivariate models. The final model included only covariates with p < 0.05. Forward stepwise methods were used. The site that was unable to identify controls was excluded from risk factor analyses. Analyses were conducted using Stata V10.0 (Texas, USA) and SAS V9.1 (Carey, NC, USA) statistical packages.

## Results

A total of 617 patients were included in this study, including 215 cancer cases and 402 controls from 13 sites (nine cancer cases were from the site that did not recruit controls). The majority (65%) of cancer cases were ADCs (n = 141); of these, 62% were NHL, 24% were KS, and 14% were cervical cancers (Table 1). The majority of KS cases were from Hong Kong (29%), Malaysia (18%), Philippines (15%), Bali (12%) and Taiwan (12%). One case was from Thailand, and there were no cases of KS reported from the India or Singapore sites (data not shown). Almost all KS cases were among men (32 of 34), of whom only 41% reported homosexual contact as mode of HIV exposure. Among NHL cases, 60% were among males, and 19% reported male homosexual contact as mode of HIV exposure.

**Table 1:** Cancer diagnosis categorized by AIDS and non-AIDS defining, and summarised by sex

	Male	Female	Total
**AIDS defining cancers (ADCs)**			
Cervix	0	20	20
Kaposi sarcoma	32	2	34
NHL - Burkitt's	8	1	9
NHL - DLBL	20	4	24
NHL - Other	34	12	46
NHL - primary of brain	7	1	8
*Subtotal*	*101*	*40*	*141*
			
**Non-AIDS defining cancers (NADCs)**			
*NADC-IUR*			
Acute lymphocytic leukaemia	1	0	1
Acute promyelocytic leukaemia	1	0	1
BCC	1	0	1
Breast	0	10	10
Cancer of unknown primary	2	1	3
Colon	2	0	2
Endometrial	0	2	2
Facial sinus tumour - NOS	1	0	1
Kidney	1	0	1
Lung	11	1	12
Multiple myeloma	2	0	2
Oesophagus	2	0	2
Pancreas	1	0	1
Plasmacytoma	2	0	2
Rectum	2	1	3
Sarcoma - NOS	0	1	1
Thyroid	3	0	3
*Subtotal*	*32*	*16*	*48*
*NADC-IR*			
Anus	2	0	2
Bladder	1	0	1
Hepatocellular carcinoma	4	1	5
Hodgkin's lymphoma - NOS	1	0	1
Hodgkin's lymphoma - depletion type	1	0	1
Hodgkin's lymphoma - mixed cellularity	1	2	3
Larynx	1	0	1
Leiomyosarcoma - EBV associated	0	2	2
Leiomyosarcoma - smooth muscle tumour	0	1	1
Nasopharynx	1	0	1
Oral cavity	1	0	1
Penile	2	0	2
Stomach	2	0	2
Tongue	2	0	2
Tonsil	1	0	1
*Subtotal*	*20*	*6*	*26*
Grand total	153	62	215

DLBL: diffuse large B cell lymphoma; EBV: Epstein-Barr virus; NOS: Not otherwise specified

Among the NADCs, 48 (22% of total cancers) were NADCs-IUR, and 26 (12% of total cancers) were NADCs-IR (probable/possible). Lung (25%) and breast cancer (21%) were the most common NADCs-IUR. Hepatocellular carcinoma (HCC) and Hodgkin's lymphoma were the most common NADCs-IR (19% each), followed by leiomyosarcoma (12%).

Patient demographics are summarized in Table 2. The majority of cancer cases were male (71%), and the mean age (SD) for the cases was 39 years (10.6) for ADCs, 46 (11.5) for NADCs-IUR and 44 (13.7) for NADCs-IR. The rate of current smoking was greater among the NADC-IR (15%), compared with 9% and 10% among the ADC and NADC-IUR groups, and 12% among the controls. Homosexual contact as mode of HIV transmission was reported by 19% of those with ADCs, compared with 6% among those with NADCs-IUR, 15% among those with NADCs-IR, and 11% among the controls. When cases of KS were excluded from the ADC group, only 13% reported homosexual exposure as mode of HIV transmission.

**Table 2:** Patient demographics at time of cancer case diagnosis for cases and controls

	ADC		NADC-IUR		NADC-IR		Controls		Total
**Total**	**141**		**48**		**26**		**402**		**617**
Male	101	71.6	32	66.7	20	76.9	279	69.4	432
Female	40	28.4	16	33.3	6	22.2	123	30.6	185
									
Mean age (SD)	39.0	(10.6)	45.7	(11.5)	44.4	(13.7)	38.8	(11.0)	39.6 (11.2)
									
Homosexual	27	19.1	3	6.3	4	15.4	43	10.7	77
Heterosexual	92	65.2	42	87.5	18	69.2	308	76.6	460
IDU	4	2.8	0	0.0	0	0.0	19	4.7	23
Other	18	12.8	3	6.3	4	15.4	32	8.0	57
									
Smoking									
Never	76	53.9	25	52.1	7	26.9	197	49.0	305
Ever	29	20.6	11	22.9	10	38.5	67	16.7	117
Current	13	9.2	5	10.4	4	15.4	47	11.7	69
Missing	23	16.3	7	14.6	5	19.2	91	22.6	126
									
Alcohol									
Never	77	54.6	29	60.4	8	30.8	218	54.2	332
Ever	25	17.7	8	16.7	6	23.1	59	14.7	98
Current	16	11.3	2	4.2	4	15.4	34	8.5	56
Missing	23	16.3	9	18.8	8	30.8	91	22.6	131
									
Caucasian	2	1.4	2	4.2	0	0.0	3	0.7	7
Chinese	48	34.0	10	20.8	8	30.8	119	29.6	185
Indian	44	31.2	15	31.3	4	15.4	132	32.8	195
Malay	4	2.8	0	0.0	0	0.0	9	2.2	13
Filipino	6	4.3	1	2.1	1	3.8	12	3.0	20
Thai	18	12.8	17	35.4	10	38.5	82	20.4	127
Indonesian	6	4.3	1	2.1	0	0.0	14	3.5	21
Vietnamese	1	0.7	0	0.0	0	0.0	0	0.0	1
Other	12	8.5	2	4.2	3	11.5	31	7.7	48
									
HBVS negative	73	51.8	24	50.0	16	61.5	232	57.7	345
HBVS positive	6	4.3	4	8.3	4	15.4	18	4.5	32
Missing	62	44.0	20	41.7	6	23.1	152	37.8	240
									
HCV negative	59	41.8	18	37.5	14	53.8	182	45.3	273
HCV positive	5	3.5	2	4.2	3	11.5	18	4.5	28
Missing	77	54.6	28	58.3	9	34.6	202	50.2	316
									
No prior AIDS	46	32.6	23	47.9	13	50.0	175	43.5	257
Prior AIDS	69	48.9	15	31.3	11	42.3	186	46.3	281
Missing	26	18.4	10	20.8	2	7.7	41	10.2	79
									
CDC A	35	24.8	15	31.3	4	15.3	109	27.1	163
CDC B	13	9.2	4	8.3	12	46.2	89	22.1	118
CDC C	89	63.1	22	45.8	9	34.6	190	47.3	310
Missing	4	2.8	7	14.6	1	3.8	14	3.5	26
									
CD4 cells/mm^3^									
<= 100	44	31.2	6	12.5	6	23.1	64	15.9	120
101-200	25	17.7	8	16.7	5	19.2	40	10.0	78
201-350	13	9.2	6	12.5	6	23.1	73	18.2	98
>= 351	16	11.3	10	20.8	5	19.2	102	25.4	133
Missing	43	30.5	18	37.5	4	15.4	123	30.6	188
									
Mean CD4 (SD)	176	(194.8)	307	(243.6)	257	(211.4)	309	(241.9)	275 (236.5)
									
ART									
Never	26	18.4	16	33.3	5	19.2	73	18.2	120
Ever	12	8.5	6	12.5	5	19.2	19	4.7	42
Current	90	63.8	24	50.0	14	53.8	294	73.1	422
Missing	13	9.2	2	4.2	2	7.7	16	4.0	33

ART: antiretroviral treatment; IDU: Injecting drug user

A larger proportion of ADC cases were of Chinese (34%) or Indian (31%) ethnicity, compared with NADC cases (21% and 31% NADC-IUR, and 31% and 15% for NADC-IR). A larger proportion of people with NADCs-IR were HCV positive (11%) compared with 4% among both ADC cases and NADC-IR cases, and 5% in the controls. A greater proportion of ADC cases had a prior AIDS diagnosis (49%) than NADC cases (31% NADC-IUR and 42% NADC-IR). Mean (SD) CD4 count was lower for ADC cases (176 cells/mm^3^: SD 195) compared with NADC cases and controls (non-infection-related cancers: 307 cells/mm^3^: SD 244; infection-related cancers: 257 cells/mm^3^: SD 211) and controls (309 cells/mm^3^: SD 242). Among the NHL cases, mean CD4 counts was highest for Burkitt's (210 cells/mm^3^: SD 119), lower for diffuse large B cell lymphoma (154 cells/mm^3^: 153) and other types (145 cells/mm^3^: SD 127), and lowest for primary NHL of the brain (77 cells/mm^3^: SD 105).

### Predictors of ADCs

In univariate analyses, homosexual contact as the mode of HIV exposure (p = 0.004), CDC stage C (p = 0.035) and CD4 cell count <100 cells/mm^3 ^(p < 0.001) were associated with ADCs. In multivariate analyses, mode of HIV exposure and CD4 cell count remained as independent predictors of ADCs. Patients who reported heterosexual contact or injecting drug use as the mode of HIV exposure were at decreased risk of cancer compared with homosexual exposure (Odds Ratio (OR) 0.35, p = 0.005 and OR: 0.17, p = 0.013, respectively), and individuals with CD4 counts above 200 cells/mm^3 ^were approximately 80% less likely to be diagnosed with an ADC (p < 0.001). After adjustment for these independent predictors, CDC stage was borderline significant (p = 0.058) (Table 3).

**Table 3:** Factors associated with ADC

	Controls	Cases	OR	95%	CI	p-value	p-overall	OR	95%	CI	p-value	p-overall
Total	264	134										
Age per 5 years	39.2 (10.9)	38.5 (9.9)	0.969	0.871	1.079	0.57						
												
Male	179	96										
Female	85	38	0.77	0.44	1.32	0.340						
												
**Homosexual**	26	25										
**Heterosexual**	202	89	**0.35**	**0.18**	**0.72**	**0.004**	**0.005**	**0.35**	**0.16**	**0.73**	**0.005**	**0.007**
**IDU**	17	4	**0.18**	**0.05**	**0.67**	**0.010**		**0.17**	**0.04**	**0.69**	**0.013**	
**Other**	19	16	**1.05**	**0.44**	**2.51**	**0.911**		**1.14**	**0.44**	**2.96**	**0.784**	
												
Chinese	82	41										
Indian	94	44	0.24	0.03	2.08	0.196	0.328					
Other	88	49	1.25	0.55	2.83	0.590						
												
No prior AIDS	106	45										
Prior AIDS	124	63	1.31	0.81	2.13	0.273	0.247					
Missing	34	26	4.25	1.52	11.89	0.006						
												
CDC A	62	25										
CDC B	54	11	0.59	0.24	1.48	0.263	0.002	0.57	0.21	1.52	0.258	0.058
CDC C	137	94	2.18	1.06	4.51	0.035		1.73	0.76	3.94	0.191	
Missing	11	4	1.26	0.31	5.10	0.746		0.75	0.16	3.57	0.713	
												
**CD4 cells/mm^3^**												
**<= 100**	47	42										
**101-200**	26	24	**0.84**	**0.41**	**1.72**	**0.637**	**<0.001**	**0.73**	**0.34**	**1.55**	**0.412**	**<0.001**
**201-350**	51	13	**0.20**	**0.09**	**0.45**	**<0.001**		**0.16**	**0.06**	**0.38**	**<0.001**	
**>= 351**	60	14	**0.20**	**0.09**	**0.43**	**<0.001**		**0.21**	**0.09**	**0.46**	**<0.001**	
**Missing**	80	41	**0.60**	**0.30**	**1.17**	**0.133**		**0.54**	**0.27**	**1.09**	**0.085**	
												
Smoking												
Never	125	70										
Ever	83	41	0.86	0.50	1.48	0.583	0.357					
Missing	56	23	0.60	0.30	1.21	0.155						
												
Alcohol												
Never	141	71										
Ever	67	40	1.22	0.70	2.13	0.475	0.271					
Missing	56	23	0.64	0.31	1.31	0.218						
												
HBVS positive	149	70										
HBVS negative	9	5	1.21	0.36	4.00	0.758	0.558					
Missing	106	59	1.34	0.78	2.28	0.288						
												
HCV positive	117	55										
HCV negative	12	5	0.93	0.31	2.75	0.890						
Missing	135	74	1.39	0.79	2.45	0.255						
												
ART												
Never	48	26										
Ever/not current	14	12	1.58	0.66	3.78	0.304	0.126					
Current	186	83	0.80	0.47	1.37	0.420						
Missing	16	13	1.86	0.67	5.18	0.235						

ART: antiretroviral treatment; IDU: Injecting drug user

We also underwent a sensitivity analysis removing the KS cases and their controls from the logistic regression. Although the risks remained broadly similar for each of the exposure groups, overall, exposure category was no longer significant, and CD4 cell count remained the only independent risk factor for ADCs (data not shown).

### Predictors of NADCs

Increasing age, a history of smoking status and not receiving antiretroviral treatment were significantly associated with increased NADC risk overall in univariate analyses (p < 0.001; p = 0.024; p < 0.001, respectively). Declining CD4 cell count was borderline significant (p = 0.056). Older age (OR: 1.39, p = 0.001) and currently receiving antiretroviral treatment (OR: 0.29, p = 0.006) remained as the only independent predictors of NADCs overall in the multivariate model.

After adjustment for these predictors, CD4 cell count and smoking status were no longer significantly associated with NADCs (p = 0.419 and 0.296, respectively) (Table 4). We also adjusted CD4 cell count for age and smoking covariates independently, and CD4 cell count remained non-significant (p = 0.398 and p = 0.090, respectively). In the regression analyses limited to the NADCs-IUR, increasing age and non-receipt of antiretroviral treatment were significant predictors in both univariate (p = 0.001 and p = 0.002) and multivariate analyses (p = 0.005 and p = 0.008) (Table 5).

**Table 4:** Factors associated with NADC (all)

	Controls	Cases	OR	95%	CI	p-value	p-overall	OR	95%	CI	p-value	p-overall
Total	138	73										
												
**Age per 5 years**	**38.0 (11.0)**	**45.0 (12.2)**	**1.41**	**1.19**	**1.66**	**<0.001**		**1.39**	**1.15**	**1.68**	**0.001**	
												
Male	100	51										
Female	38	22	1.07	0.52	2.21	0.853						
												
Homosexual	17	7	1.00									
Heterosexual	106	59	1.51	0.46	4.92	0.494	0.926					
IDU	2	0	0.00	0.00	.	0.992						
Other	13	7	1.45	0.31	6.76	0.635						
												
Chinese	37	17										
Indian*	38	19	--	--	--	--						
Other	63	37	1.71	0.41	7.05	0.458						
												
No prior AIDS	69	35										
Prior AIDS	62	26	0.98	0.52	1.83	0.946	0.787					
Missing	7	12	9.15	1.88	44.61	0.006						
												
CDC A	44	17										
CDC B	34	14	1.13	0.45	2.85	0.799	0.179					
CDC C	56	33	1.70	0.74	3.91	0.214						
Missing	4	9	7.04	1.69	29.25	0.007						
												
												
CD4 cells/mm^3^												
<= 100	17	12	1.00									
101-200	14	13	1.12	0.38	3.34	0.839	0.056	1.08	0.27	4.38	0.915	0.419
201-350	22	12	0.55	0.17	1.78	0.316		0.91	0.24	3.41	0.888	
>= 351	42	15	0.37	0.13	1.09	0.072		0.57	0.17	1.94	0.368	
Missing	43	21	0.64	0.25	1.67	0.366		0.78	0.25	2.45	0.667	
												
Smoking												
Never	72	31	1.00									
Ever	31	29	2.32	1.13	4.76	0.022	0.024	1.68	0.72	3.93	0.231	0.296
Missing	35	13	0.75	0.29	1.92	0.548		0.69	0.20	2.36	0.559	
												
Alcohol												
Never	77	36	1.00									
Ever	26	19	1.61	0.77	3.37	0.210	0.405					
Missing	35	18	0.95	0.41	2.19	0.900						
												
HBVS negative	83	40	1.00									
HBVS positive	9	8	1.91	0.66	5.54	0.234	0.451					
Missing	46	25	1.29	0.61	2.70	0.503						
												
HCV negative	65	32	1.00									
HCV positive	6	5	1.68	0.43	6.62	0.459	0.654					
Missing	67	36	1.32	0.62	2.83	0.473						
												
**ART**												
**Never**	25	21	**1.00**									
**Ever/not current**	5	11	**1.92**	**0.51**	**7.20**	**0.330**	**<0.001**	**1.42**	**0.35**	**5.77**	**0.625**	**0.001**
**Current**	108	37	**0.28**	**0.12**	**0.65**	**0.003**		**0.29**	**0.12**	**0.70**	**0.006**	
**Missing**	0	4	**- -**	**- -**	**- -**	**- -**	**- -**	**- -**	**- -**	**- -**	**- -**	

ART: antiretroviral treatment; IDU: Injecting drug user

**Table 5:** Factors associated with NADC-IUR

	Controls	Cases	OR	95%	CI	p-value	p-overall	OR	95%	CI	p-value	p-overall
Total	91	47										
												
**Age per 5 years**	**37.9 (10.6)**	**45.3 (11.4)**	**1.40**	**1.15**	**1.70**	**0.001**		**1.36**	**1.10**	**1.69**	**0.005**	
												
Male												
Female			1.58	0.62	4.02	0.337						
												
Homosexual	6	3										
Heterosexual	77	41	1.00	0.18	5.46	1.000	0.987					
IDU	1	0	--	--	--	--						
Other	7	3	0.75	0.08	7.24	0.806						
												
Chinese	21	9										
Indian	30	15	2.00	0.40	9.91	0.396						
Other	40	23										
												
No prior AIDS	42	22										
Prior AIDS	44	15	0.75	0.35	1.64	0.474	0.390					
Missing	5	10	13.35	1.58	112.58	0.017						
												
CDC A	21	14										
CDC B	27	4	0.12	0.02	0.62	0.011	0.775					
CDC C	40	22	0.37	0.09	1.52	0.170						
Missing	3	7	2.66	0.44	16.06	0.286						
												
CD4 cells/mm^3^												
<= 100	15	6	2.17	0.54	8.66	0.272	0.837					
101-200	9	8	1.07	0.26	4.49	0.925						
201-350	12	6	0.97	0.26	3.64	0.968						
>= 351	23	10	1.36	0.42	4.37	0.603						
Missing	32	17										
												
Smoking												
Never	50	25	1.36	0.55	3.37	0.504	0.573					
Ever	23	15	0.71	0.22	2.30	0.564						
Missing	18	7										
												
Alcohol												
Never	56	29	0.95	0.35	2.62	0.922	0.991					
Ever	18	9	0.94	0.31	2.88	0.916						
Missing	17	9										
												
HBVS negative	51	24										
HBVS positive	7	4	1.31	0.33	5.24	0.707	0.713					
Missing	33	19	1.43	0.59	3.44	0.430						
												
HCV negative	41	18										
HCV positive	3	2	1.33	0.11	15.73	0.820	0.535					
Missing	47	27	1.76	0.65	4.75	0.263						
												
**ART**												
**Never**	**16**	**16**										
**Ever/not current**	**5**	**6**	**1.00**	**0.23**	**4.28**	**0.999**	**0.002**	**0.81**	**0.17**	**3.86**	**0.788**	**0.008**
**Current**	**70**	**23**	**0.23**	**0.09**	**0.62**	**0.004**		**0.26**	**0.09**	**0.74**	**0.012**	
**Missing**	**0**	**2**	**- -**	**- -**	**- -**	**- -**		**- -**	**- -**	**- -**	**- -**	

ART: antiretroviral treatment; IDU: Injecting drug user

Due to the small numbers, covariates assessed for association with NADCs-IR were limited to factors that were significant in univariate analyses for either the ADC or NADC overall endpoints. These included age, CDC stage, CD4 cell count, smoking status and antiretroviral treatment. As HCC was one of the most common NADCs-IR, and all five cases were either HBV surface antigen (n = 4) or HCV core antibody positive (n = 3), we also assessed coinfection with HBV or HCV. In univariate analyses, increasing age (p = 0.023), increasing CDC stage (p = 0.032), CD4 category >200 cells/mm^3 ^(p = 0.041), and ever smoking (p = 0.020) were associated with NADCs-IR (Table 6). CD4 cell count and CDC stage remained significant in the multivariate analyses (p = 0.041 each). In the sensitivity analysis excluding bladder, larynx and oral cavity cancers (n = 3), the results remained largely unchanged (data not shown).

**Table 6:** Factors associated with NADC-IR

	Controls	Cases	OR	95%	CI	p-value	p-overall	OR	95%	CI	p-value	p-overall
Age per 5 years			1.43	1.05	1.94	0.023						
												
HCV core antibody												
Negative	32	16										
Positive	2	4	1.64	0.30	8.94	0.566	0.206					
Missing	13	6	0.82	0.23	2.89	0.756						
												
HBV surface antigen												
Negative	24	14	3.26	0.55	19.25	0.192	0.454					
Positive	3	3	1.03	0.25	4.21	0.972						
Missing	20	9										
												
**CDC A**	**23**	**3**										
**CDC B**	**7**	**10**	**9.29**	**1.74**	**49.63**	**0.009**	**0.032**	**32.72**	**2.22**	**481.07**	**0.011**	**0.041**
**CDC C**	**16**	**11**	**5.96**	**1.11**	**32.13**	**0.038**		**12.14**	**0.84**	**175.11**	**0.067**	
**Missing**	**1**	**2**	**13.50**	**0.81**	**223.93**	**0.069**		**22.30**	**0.54**	**913.42**	**0.101**	
												
**CD4 cells/mm^3^**												
**<= 100**	**2**	**6**										
**101-200**	**5**	**5**	**0.26**	**0.02**	**2.85**	**0.271**	**0.021**	**0.79**	**0.04**	**14.43**	**0.876**	**0.041**
**201-350**	**10**	**6**	**0.05**	**0.00**	**0.94**	**0.045**		**0.03**	**0.00**	**2.15**	**0.108**	
**>= 351**	**19**	**5**	**0.03**	**0.00**	**0.44**	**0.011**		**0.01**	**0.00**	**0.48**	**0.022**	
**Missing**	**11**	**4**	**0.05**	**0.00**	**0.89**	**0.041**		**0.04**	**0.00**	**1.38**	**0.075**	
												
ART												
Never	9	5	0.65	0.13	3.16	0.595						
Ever	38	19	--	--	--	--						
Missing	0	2										
												
Smoking												
Never	22	6										
Ever	8	14	6.06	1.52	24.21	0.011	0.020					
Missing	17	6	0.95	0.20	4.66	0.953						

ART: antiretroviral treatment

## Discussion

In this retrospective case-control study, more than half (66%) the cancer cases identified were ADCs. The remaining cancers were either NADCs-IUR (22%) or NADCs-IR (12%). NHL was the most commonly reported ADC overall, as well as among men, while cervical cancer was the most common among women. Lung and breast cancers were the most commonly reported NADCs overall, and hepatocellular carcinoma was the most common NADC-IR. Factors associated with ADCs were immunodeficiency and lower CD4 cell count, while among NADCs overall and for NADCs-IUR, factors were older age and not currently receiving antiretroviral treatment. CDC stage C and lower CD4 cell count were significantly associated with NADCs-IR.

A novel finding in our study was the reporting of KS cases. KS has been thought to be relatively rare in some countries of Asia, largely attributed to the low prevalence of human herpes virus (HHV8) known to cause KS [33,34]. KS cases were largely reported from the Hong Kong site, and from Malaysia, the Philippines and Taiwan. In the Thai study, KS was reported in only 5% of ADCs [26], and no cases of KS were observed in the Mumbai study [27]. Almost all the KS cases in our study were among men (94%), of whom only 41% reported homosexual contact as the mode of HIV transmission. In western countries, KS occurs predominately among homosexual men [21]. We believe that our findings may reflect underreporting of male-to-male sex as a primary or concomitant risk factor for HIV infection in Asian countries.

The increased incidence of NADCs has been extensively reported in the literature [5,8,35-37]. Specific NADCs that have been reported to be higher in HIV-positive people than in HIV-negative people include Hodgkin's lymphoma, lung cancer, hepatocellular carcinoma [38], anal, vaginal, oropharyngeal, colorectal cancers, melanoma and leukemia [39], and cancer of the lips and testis [7].

Even in developing countries, where HAART is largely unavailable, the incidence of NADCs has increased. In India, there has been an increase in anal cancer, Hodgkin's lymphoma, testicular and colon cancers, and head and neck cancers [27]. In our study, lung and breast cancers were the most commonly reported NADCs, as well as head and neck cancers. Although lung cancer has been identified as one of several NADCs at increased incidence in HIV-infected patients, for breast cancer, the evidence of increasing incidence is still equivocal [31,35,40,41]. In the Thai study, the prevalence of breast cancer was 3%, and 10% of all NADCs, a little lower than our 14% of NADCs [26]. Head and neck cancers have been reported as the most common NADC in one Indian study [27].

Among the NADCs-IR, HCC was the most frequently reported. HCC is a commonly reported NADC in the literature, and is likely to remain important in HIV-infected populations, particularly in the context of coinfection with HBV and HCV [22]. In our study, all five cases were either HCV or HBV positive. Although we did not find a statistically significant association of HCV or HBV coinfection and NADCs-IR, this may explained by low numbers, but may also likely be due to the inclusion of other cancers in this analysis, whose primary risk is not HBV or HCV infection.

Also of particular interest were the three cases of leiomyosarcoma reported, all in women. Leiomyosarcoma, smooth-muscle tumours, are usually seen in children and young adults with AIDS [42], yet overall are quite rare. Epstein-Barr virus has been associated with this tumour, and only sporadic case reports have been published [43].

### Factors associated with ADCs and NADCs

Immune suppression and exposure category were independently associated with ADCs in our study, similar to what has been reported in the literature [3,39]. The risk of an ADC decreased by approximately 80% with CD4 cell counts >200 cells/mm^3^, while patients who reported homosexual contract as the mode of HIV infection had an almost three-fold increased risk of an ADC compared with those who reported heterosexual contact, and six-fold increased risk compared with those who reported injecting drug use.

However, the exposure category effect we observed is largely explained by a greater proportion of KS cases reporting homosexual contact. In the sensitivity analyses where KS cases were excluded, the homosexual exposure effect was removed, and immunodeficiency remained the only significant factor. A substantial proportion of patients in this study were diagnosed with HIV at the time of the cancer diagnosis, with more than a quarter of cancers diagnosed within one month of HIV diagnosis (28% and 26% of ADCs and NADCs, respectively). Late diagnosis may reflect particular marginalized groups, such as men who have sex with men, and their reluctance to seek medical care.

Older age and having never received antiretroviral treatment, but not CD4 count, were associated with being diagnosed with any NADC, both overall and for NADC-IUR, similar to other studies [3,37]. Longer duration of HIV infection and a history of opportunistic infections have also been reported [35].

In our study, immune suppression (CDC stage and lower CD4 cell count) was associated with NADCs-IR. Several studies examining CD4 and NADCs during the HAART era have not found an association with CD4 [3,7,37]. However, these studies did not restrict their analysis to NADCs that are currently thought to be infection related. More recent data have shown several NADCs in HIV-positive patients to be increasing, similar to the risk seen in transplantation recipients, suggesting a link between immune suppression and increased risk of a specific NADC [29].

Smoking was not associated with NADCs-IUR, despite lung cancer being the most commonly cancer reported in this group, and yet a mild association was observed for NADCs-IR. We believe the lack of association with the NADC-IUR group is due largely to the inclusion of cancers where smoking is not a primary risk factor. The association with smoking and NADCs-IR on the other hand may reflect risk behaviour, where people who may engage in unprotected sex or drug use, which lead to viral coinfections, may also be more likely to smoke.

There are some limitations to this study. First, this is a retrospective survey of cancer cases and controls, and subject to selection bias and concerns regarding complete ascertainment of cases. Most sites reviewed all patient records for case ascertainment since 2000, while some sites reviewed more recent time periods or restricted review to TAHOD patients. Furthermore, there may be variation between TAHOD sites in the level of cancer screening, despite sites being primarily urban referral centres. Care must be taken in generalizing our results to Asian patients in other types of clinical centres or geographic locales.

Second, our division between NADCs-IUR and NADCs-IR may be debated. Included in our list of NADCs-IR were cancers where only a small proportion (≤20%) were attributed to infections (including bladder, larynx and oral cavity). However, we did conduct a predictor analysis for NADCs overall, as well as dividing cases by relationship to infection, and our results were consistent with what has been shown previously in the literature. Finally, we did not have enough NADC cases to examine patient characteristics and factors associated with individual cancers potentially masking risk factors for specific cancers, such as that demonstrated by smoking and lung cancer. Nor did we collect any other behavioural data beyond smoking and alcohol use.

## Conclusions

In conclusion, the spectrum of cancer diagnoses in the Asia region currently does not appear dissimilar to that observed in non-Asian HIV populations. Key findings in our study include the much more commonly diagnosed KS than expected, as KS has been widely thought to be rare in Asia. This may reflect a common misreporting of HIV exposure among men in Asia. Second, we identified three cases of leiomyosarcoma, a rare and uncommonly reported malignancy, with links to Epstein-Barr virus.

Third, this is the first study to our knowledge to have examined both infection-related and infection-unrelated NADCs in the Asia region. Given the diversity in prevalence and infectious agents between countries within this region, further detailed studies are required to better describe the range of cancers in this region. To this end, new diagnoses of cancers have been prospectively reported in TAHOD since 2008. These studies will help guide where screening programmes are needed.

## Competing interests

The authors declare that they have no competing interests.

## Authors' contributions

The TAHOD Steering Committee was responsible for the overall design of the TAHOD Retrospective Cancer Study. KP, ML and AG designed the current study concept. KP conducted the statistical analyses and drafted the manuscript. All members of the writing committee (KP, EH, NK, SK, JC, YC, TM, FJ, PL, SS, SP, SP, RD, CL, AG and ML) discussed the analysis plan, contributed to interpretation of the analysis results and commented on drafts of the manuscript. All authors approved the final manuscript draft for journal submission.

## References

[B1] Frisch M Biggar RJ Engels EA Goedert JJ Association of cancer with AIDS-related immunosuppression in adults Jama 2001 13 1736 1745 10.1001/jama.285.13.1736 11277828

[B2] Goedert JJ Cote TR Virgo P Scoppa SM Kingma DW Gail MH Jaffe ES Biggar RJ Spectrum of AIDS-associated malignant disorders Lancet 1998 13 1833 1839 10.1016/S0140-6736(97)09028-4 9652666

[B3] Mbulaiteye SM Biggar RJ Goedert JJ Engels EA Immune deficiency and risk for malignancy among persons with AIDS J Acquir Immune Defic Syndr 2003 13 527 533 10.1097/00126334-200304150-00010 12679705

[B4] Centers for Disease Control and Prevention 1993 revised classification system for HIV infection and expanded surveillance case definition for AIDS among adolescents and adults Morbidity and Mortality Weekly Report 1992 13 RR 17 1361652

[B5] Hessol NA Pipkin S Schwarcz S Cress RD Bacchetti P Scheer S The Impact of Highly Active Antiretroviral Therapy on Non-AIDS-Defining Cancers among Adults with AIDS Am J Epidemiol 2007 13 1143 1153 10.1093/aje/kwm017 17344204

[B6] Franceschi S Dal Maso L Arniani S Lo Re A Barchielli A Milandri C Simonato L Vercelli M Zanetti R Rezza G Linkage of AIDS and cancer registries in Italy Int J Cancer 1998 13 831 834 10.1002/(SICI)1097-0215(19980316)75:6<831::AID-IJC3>3.0.CO;2-W 9506526

[B7] Grulich AE Li Y McDonald A Correll PK Law MG Kaldor JM Rates of non-AIDS-defining cancers in people with HIV infection before and after AIDS diagnosis Aids 2002 13 1155 1161 10.1097/00002030-200205240-00009 12004274

[B8] Engels EA Pfeiffer RM Goedert JJ Virgo P McNeel TS Scoppa SM Biggar RJ Trends in cancer risk among people with AIDS in the United States 1980-2002 Aids 2006 13 1645 1654 10.1097/01.aids.0000238411.75324.59 16868446

[B9] Clifford GM Polesel J Rickenbach M Dal Maso L Keiser O Kofler A Rapiti E Levi F Jundt G Fisch T Bordoni A De Weck D Franceschi S Cancer risk in the Swiss HIV Cohort Study: associations with immunodeficiency, smoking, and highly active antiretroviral therapy J Natl Cancer Inst 2005 13 425 432 10.1093/jnci/dji072 15770006

[B10] Newnham A Harris J Evans HS Evans BG Moller H The risk of cancer in HIV-infected people in southeast England: a cohort study Br J Cancer 2005 13 194 200 10.1038/sj.bjc.6602273 15583689 PMC2361741

[B11] International Collaboration on HIV and Cancer Highly active antiretroviral therapy and incidence of cancer in human immunodeficiency virus-infected adults J Natl Cancer Inst 2000 13 1823 1830 10.1093/jnci/92.22.1823 11078759

[B12] Chiao EY Krown SE Update on non-acquired immunodeficiency syndrome-defining malignancies Curr Opin Oncol 2003 13 389 397 10.1097/00001622-200309000-00008 12960522

[B13] Mocroft A Vella S Benfield TL Chiesi A Miller V Gargalianos P d'Arminio Monforte A Yust I Bruun JN Phillips AN Lundgren JD Changing patterns of mortality across Europe in patients infected with HIV-1. EuroSIDA Study Group Lancet 1998 13 1725 1730 10.1016/S0140-6736(98)03201-2 9848347

[B14] Mocroft A Brettle R Kirk O Blaxhult A Parkin JM Antunes F Francioli P D'Arminio Monforte A Fox Z Lundgren JD Changes in the cause of death among HIV positive subjects across Europe: results from the EuroSIDA study Aids 2002 13 1663 1671 10.1097/00002030-200208160-00012 12172088

[B15] Cohen MH French AL Benning L Kovacs A Anastos K Young M Minkoff H Hessol NA Causes of death among women with human immunodeficiency virus infection in the era of combination antiretroviral therapy Am J Med 2002 13 91 98 10.1016/S0002-9343(02)01169-5 12133746 PMC3126666

[B16] Jain MK Skiest DJ Cloud JW Jain CL Burns D Berggren RE Changes in mortality related to human immunodeficiency virus infection: comparative analysis of inpatient deaths in 1995 and in 1999-2000 Clin Infect Dis 2003 13 1030 1038 10.1086/368186 12684916

[B17] Palella FJ Delaney KM Moorman AC Loveless MO Fuhrer J Satten GA Aschman DJ Holmberg SD Declining morbidity and mortality among patients with advanced human immunodeficiency virus infection. HIV Outpatient Study Investigators N Engl J Med 1998 13 853 860 10.1056/NEJM199803263381301 9516219

[B18] Chaisson RE Keruly JC Moore RD Association of initial CD4 cell count and viral load with response to highly active antiretroviral therapy Jama 2000 13 3128 3129 10.1001/jama.284.24.3128 11135775

[B19] Mocroft A Ledergerber B Katlama C Kirk O Reiss P d'Arminio Monforte A Knysz B Dietrich M Phillips AN Lundgren JD Decline in the AIDS and death rates in the EuroSIDA study: an observational study Lancet 2003 13 22 29 10.1016/S0140-6736(03)13802-0 12853195

[B20] Correll PK Law MG McDonald AM Cooper DA Kaldor JM HIV disease progression in Australia in the time of combination antiretroviral therapies Med J Aust 1998 13 469 472 9847898

[B21] Grulich AE Li Y McDonald AM Correll PK Law MG Kaldor JM Decreasing rates of Kaposi's sarcoma and non-Hodgkin's lymphoma in the era of potent combination anti-retroviral therapy AIDS 2001 13 629 633 10.1097/00002030-200103300-00013 11317001

[B22] Bonnet F Lewden C May T Heripret L Jougla E Bevilacqua S Costagliola D Salmon D Chene G Morlat P Malignancy-related causes of death in human immunodeficiency virus-infected patients in the era of highly active antiretroviral therapy Cancer 2004 13 317 324 10.1002/cncr.20354 15241829

[B23] Cheung MC Pantanowitz L Dezube BJ AIDS-related malignancies: emerging challenges in the era of highly active antiretroviral therapy Oncologist 2005 13 412 426 10.1634/theoncologist.10-6-412 15967835

[B24] Petoumenos K Law MG Risk factors and causes of death in the Australian HIV Observational Database Sex Health 2006 13 103 112 10.1071/SH05045 16800396

[B25] D'Arminio Monforte A Abrams D Pradier C Weber R Bonnet F de Wit S Friis-Moller N Phillips A Sabin C Lundgren J The D:A:D Study Group HIV-induced immunodeficiency and risk of fatal AIDS-defining and non-AIDS defining malignancies: Results from the D:A:D Study CROI 2007 Abstract No: 84 10.1097/QAD.0b013e3283112b77 PMC2715844 18832878

[B26] Kiertiburanakul S Likhitpongwit S Ratanasiri S Sungkanuparph S Malignancies in HIV-infected Thai patients HIV Med 2007 13 322 323 10.1111/j.1468-1293.2007.00471.x 17561879

[B27] Dhir AA Sawant S Dikshit RP Parikh P Srivastava S Badwe R Rajadhyaksha S Dinshaw KA Spectrum of HIV/AIDS related cancers in India Cancer Causes Control 2008 13 147 153 10.1007/s10552-007-9080-y 17992576

[B28] Vajdic CM McDonald SP McCredie MR van Leeuwen MT Stewart JH Law M Chapman JR Webster AC Kaldor JM Grulich AE Cancer incidence before and after kidney transplantation Jama 2006 13 2823 2831 10.1001/jama.296.23.2823 17179459

[B29] Grulich AE van Leeuwen MT Falster MO Vajdic CM Incidence of cancers in people with HIV/AIDS compared with immunosuppressed transplant recipients: a meta-analysis Lancet 2007 13 59 67 10.1016/S0140-6736(07)61050-2 17617273

[B30] Zhou J Kumarasamy N Ditangco R Kamarulzaman A Lee CK Li PC Paton NI Phanuphak P Pujari S Vibhagool A Wong WW Zhang F Chuah J Frost KR Cooper DA Law MG The TREAT Asia HIV Observational Database: baseline and retrospective data J Acquir Immune Defic Syndr 2005 13 174 179 10.1097/01.qai.0000145351.96815.d5 15671802 PMC3070962

[B31] Silverberg MJ Chao C Leyden WA Xu L Tang B Horberg MA Klein D Quesenberry CP Jr Towner WJ Abrams DI HIV infection and the risk of cancers with and without a known infectious cause AIDS 2009 13 2337 2345 10.1097/QAD.0b013e3283319184 19741479 PMC2863991

[B32] Parkin DM The global health burden of infection-associated cancers in the year 2002 Int J Cancer 2006 13 3030 3044 10.1002/ijc.21731 16404738

[B33] Ablashi D Chatlynne L Cooper H Thomas D Yadav M Norhanom AW Chandana AK Churdboonchart V Kulpradist SA Patnaik M Liegmann K Masood R Reitz M Cleghorn F Manns A Levine PH Rabkin C Biggar R Jensen F Gill P Jack N Edwards J Whitman J Boshoff C Seroprevalence of human herpesvirus-8 (HHV-8) in countries of Southeast Asia compared to the USA, the Caribbean and Africa Br J Cancer 1999 13 893 897 10.1038/sj.bjc.6690782 10555764 PMC2374301

[B34] Ayuthaya PI Katano H Inagi R Auwanit W Sata T Kurata T Yamanishi K The seroprevalence of human herpesvirus 8 infection in the Thai population Southeast Asian J Trop Med Public Health 2002 13 297 305 12236428

[B35] Burgi A Brodine S Wegner S Milazzo M Wallace MR Spooner K Blazes DL Agan BK Armstrong A Fraser S Crum NF Incidence and risk factors for the occurrence of non-AIDS-defining cancers among human immunodeficiency virus-infected individuals Cancer 2005 13 1505 1511 10.1002/cncr.21334 16104038

[B36] Bedimo R Chen RY Accortt NA Raper JL Linn C Allison JJ Dubay J Saag MS Hoesley CJ Trends in AIDS-defining and non-AIDS-defining malignancies among HIV-infected patients: 1989-2002 Clin Infect Dis 2004 13 1380 1384 10.1086/424883 15494916

[B37] Crum-Cianflone N Hullsiek KH Marconi V Weintrob A Ganesan A Barthel RV Fraser S Agan BK Wegner S Trends in the incidence of cancers among HIV-infected persons and the impact of antiretroviral therapy: a 20-year cohort study AIDS 2009 13 41 50 10.1097/QAD.0b013e328317cc2d 19050385 PMC2727153

[B38] Engels EA Biggar RJ Hall HI Cross H Crutchfield A Finch JL Grigg R Hylton T Pawlish KS McNeel TS Goedert JJ Cancer risk in people infected with human immunodeficiency virus in the United States Int J Cancer 2008 13 187 194 10.1002/ijc.23487 18435450

[B39] Patel P Hanson DL Sullivan PS Novak RM Moorman AC Tong TC Holmberg SD Brooks JT Incidence of types of cancer among HIV-infected persons compared with the general population in the United States, 1992-2003 Ann Intern Med 2008 13 728 736 18490686 10.7326/0003-4819-148-10-200805200-00005

[B40] Pantanowitz L Dezube BJ Breast cancer in HIV positive women: a report of four cases and review of the literature Cancer Invest 2003 13 665 10.1081/CNV-120022389 14533455

[B41] Pantanowitz L Dezube BJ Reasons for a deficit of breast cancer among HIV-infected patients J Clin Oncol 2004 13 1347 1348 author reply 1349-1350 10.1200/JCO.2004.99.261 15051793

[B42] Linos D Kiriakopoulos AC Tsakayannis DE Theodoridou M Chrousos G Laparoscopic excision of bilateral primary adrenal leiomyosarcomas in a 14-year-old girl with acquired immunodeficiency syndrome (AIDS) Surgery 2004 13 1098 1100 10.1016/j.surg.2003.07.007 15523410

[B43] Zevallos-Giampietri EA Yanes HH Orrego Puelles J Barrionuevo C Primary meningeal Epstein-Barr virus-related leiomyosarcoma in a man infected with human immunodeficiency virus: review of literature, emphasizing the differential diagnosis and pathogenesis Appl Immunohistochem Mol Morphol 2004 13 387 391 15536343 10.1097/00129039-200412000-00018

